# Exact solving and sensitivity analysis of stochastic continuous time Boolean models

**DOI:** 10.1186/s12859-020-03548-9

**Published:** 2020-06-11

**Authors:** Mihály Koltai, Vincent Noel, Andrei Zinovyev, Laurence Calzone, Emmanuel Barillot

**Affiliations:** 1 Institut Curie, PSL Research University, Paris, F-75005 France; 2 INSERM, U900, Paris, F-75005 France; 3 CBIO-Centre for Computational Biology, MINES ParisTech, PSL Research University, Paris, F-75006 France

**Keywords:** Boolean modeling, Exact method, Stochastic model, Asynchronous updating, Steady state solution, Continuous time Markov chain

## Abstract

**Background:**

Solutions to stochastic Boolean models are usually estimated by Monte Carlo simulations, but as the state space of these models can be enormous, there is an inherent uncertainty about the accuracy of Monte Carlo estimates and whether simulations have reached all attractors. Moreover, these models have timescale parameters (transition rates) that the probability values of stationary solutions depend on in complex ways, raising the necessity of parameter sensitivity analysis. We address these two issues by an exact calculation method for this class of models.

**Results:**

We show that the stationary probability values of the attractors of stochastic (asynchronous) continuous time Boolean models can be exactly calculated. The calculation does not require Monte Carlo simulations, instead it uses graph theoretical and matrix calculation methods previously applied in the context of chemical kinetics. In this version of the asynchronous updating framework the states of a logical model define a continuous time Markov chain and for a given initial condition the stationary solution is fully defined by the right and left nullspace of the master equation’s kinetic matrix. We use topological sorting of the state transition graph and the dependencies between the nullspaces and the kinetic matrix to derive the stationary solution without simulations. We apply this calculation to several published Boolean models to analyze the under-explored question of the effect of transition rates on the stationary solutions and show they can be sensitive to parameter changes. The analysis distinguishes processes robust or, alternatively, sensitive to parameter values, providing both methodological and biological insights.

**Conclusion:**

Up to an intermediate size (the biggest model analyzed is 23 nodes) stochastic Boolean models can be efficiently solved by an exact matrix method, without using Monte Carlo simulations. Sensitivity analysis with respect to the model’s timescale parameters often reveals a small subset of all parameters that primarily determine the stationary probability of attractor states.

## Background

One of the principle aims of systems biology is to understand with the help of models the complex molecular networks that regulate the functioning of a cell [1]. To do so, numerous mathematical and computational formalisms have been used in the past decades [2]. These range from quantitative and mechanistic models that require the knowledge of numerous biophysical constants [3] to higher level, more qualitative models such as fuzzy logic [4] and Boolean [5, 6] models that describe functional dependencies, but not the details of biophysical mechanisms. Boolean models, originally introduced in the systems biology field by Kauffman [7–9], have the advantage that interactions between a model’s variables (that can be genes, proteins or other cellular components and their states) only need to be qualitatively defined and identifying attractors is a fast calculation [10]. Traditionally, Boolean modeling has been used as a more qualitative approach to quickly identify the stationary states (attractors) of a model and test their robustness to initial conditions and/or perturbations. In most Boolean modeling platforms [10–12], time is described in discrete steps and model outputs are binary.

In recent years, efforts were made to bridge the gap between qualitative and quantitative modeling by a continuous time stochastic version of Boolean modeling [13, 14].

With this hybrid approach, we obtain continuous values for both transient and stationary probabilities of the states of a Boolean model, while having fewer parameters than fully detailed models of chemical kinetics. For introducing continuous probability values and physical time, it is necessary to define timescale parameters (*transition rates*, defined in *Methods* below) for the activation or deactivation of the network nodes. With these timescale parameters a kinetic Monte Carlo (Gillespie) algorithm [15–17] is used in the MaBoSS modeling environment to simulate large logical models (examples include models of over a 100 nodes [18]).

The Gillespie algorithm is based on generating sample trajectories. With increasing model size and hence slower convergence to attractors the duration of simulations need to be increased, and as a compensation the number of sample trajectories is typically decreased in the interest of computation speed. Besides compromising the accuracy of probability estimates, this raises the issue of attractor reachability. As the number of states of a Boolean model of *n* nodes is 2^*n*^, stochastic simulations with a limited number of samples might not reach all attractors, leaving an uncertainty if the model’s behavior is fully explored.

Another question that needs to be addressed is parameter dependence. The probability value of an attractor state depends on the timescale parameters in a complex way. In the studies using the continuous time stochastic Boolean formalism, transition rates are usually assigned a default value (typically 1) for simplicity. However, transition rates are not neutral parameters: as shown in [18], specifying transition rates from expression data can improve a model’s predictive performance. This suggests that systematic parameter sensitivity analysis, similarly to ordinary differential equation (ODE) models [19, 20], can provide valuable insights on the key parameters of a model. For instance, in the case of a model with the outputs of proliferation versus cell death we can ask the question if there are a few transition rates that dominate this decision point.

We provide here an exact method to calculate the stationary solutions of stochastic continuous time Boolean models, adopting mathematical techniques previously applied in deterministic chemical kinetics [21, 22]. The sparsity of the transition/kinetic matrix of Boolean models is exploited, so that the calculation is as fast or faster than stochastic simulations up to an intermediate size, while being exact.

The method finds all states of the stationary solutions and their stationary probability values. We perform parameter sensitivity analysis and visualizations on a number of published Boolean models to explore how sensitive these models are to variations in transition rates.

In some cases, (local) parameter sensitivity analysis reveals that a model’s behavior is controlled by only a few transition rates, enabling model reduction and/or reducing the parameter space for more extensive (global) sensitivity analysis and parameter fitting. Based on these results, we propose that parameter sensitivity analysis should be a part of the construction of a stochastic Boolean model if a detailed mechanistic understanding is important. We provide the MATLAB toolbox *ExaStoLog* as a tool to analyze user-defined logical models. The toolbox contains the core calculation method along with visualization and sensitivity analysis tools and is available on GitHub with a detailed tutorial [23].

In the “Methods” section we first provide the basic concepts for Boolean networks in general (‘Definitions’), and for the asynchronous update policy (‘Asynchronous updating’) and the continuous time treatment (‘Continuous-time treatment of Boolean dynamics’) of Boolean models in particular. Following that we derive the exact solution of Boolean models in this stochastic continuous time framework (‘Derivation’).

## Methods

### Definitions and notations: Boolean networks

We define Boolean networks (BN) as consisting of a set of binary-valued variables called *nodes*, *V*, and a vector of Boolean functions (logical rules) *F* [24, 25]:

1 $$ V = \left\{v_{1},v_{2},...,v_{n}\right\}, \qquad F = \left\{f_{1},f_{2},...,f_{n}\right\}  $$

**Fig. 1:**
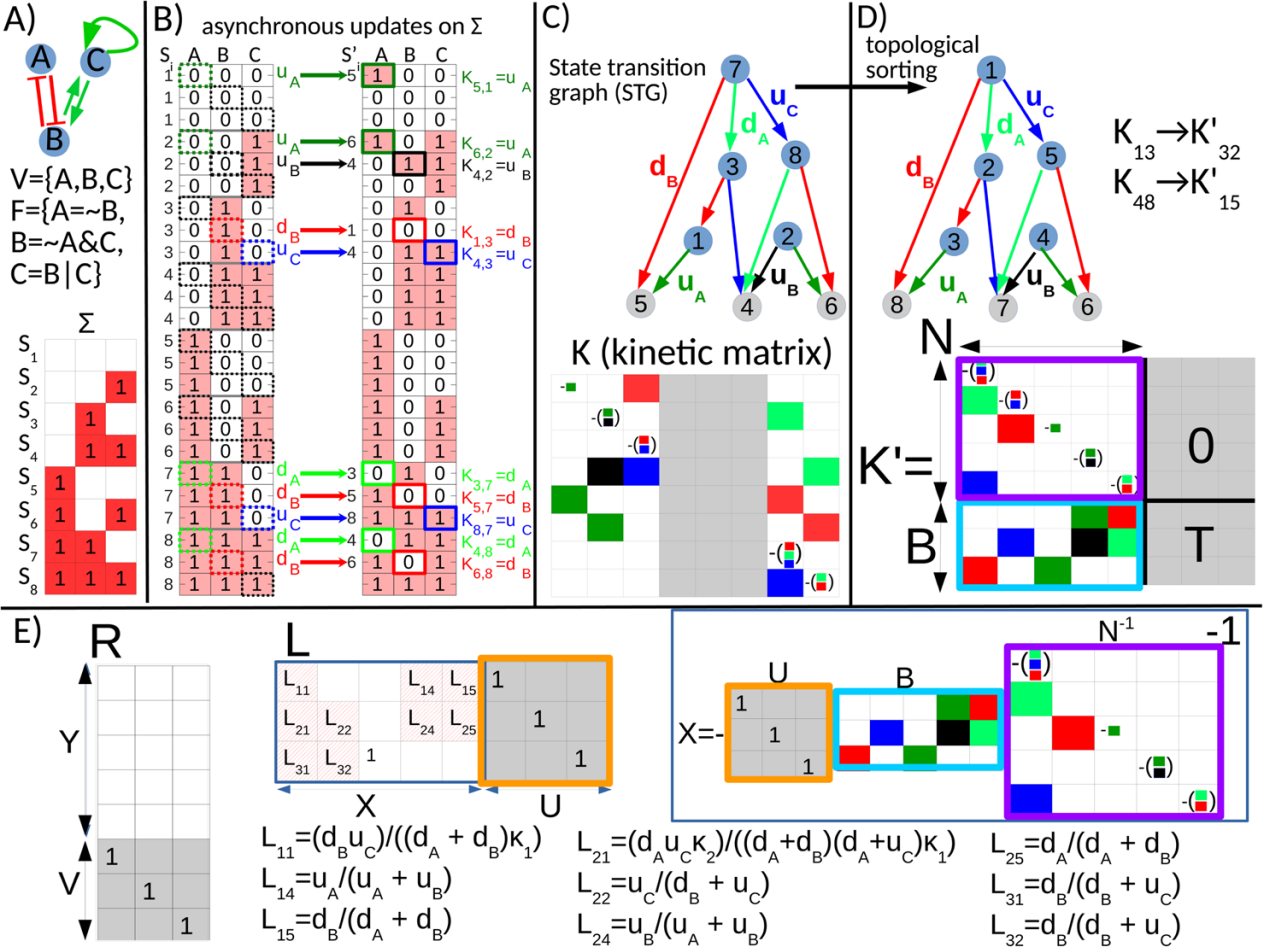
Graphical illustration of the exact calculation method for a 3-node Boolean network (BN). **a** Influence graph, list of *n*=3 nodes (*V*), Boolean functions (*F*) and state space (*Σ*) of the BN. States (*S*) within *Σ* have a decimal index, *S*_1_...*S*_8_**b** Generating the elements of the kinetic matrix by asynchronous updating. In asynchronous updating only one node of the BN changes its value at a given timepoint. This means from any state *S* there are *n* possible transitions, therefore each state *S*_*i*_ is repeated *n*=3 times. The table on the left shows the source states (*S*_*i*_) of all possible *n* 2^*n*^=24 transitions. For each state (decimal indices on the left) the Boolean functions *F* are applied individually to the three nodes, updated node highlighted by the dashed black line. If there is a change in the node’s value, the dashed square is highlighted by the color of the transition’s rate, shown next to the arrow to the right representing the BN’s transitions. If there is no change in the node’s value, there is no transition (no arrow). The table on the right shows the target states (*S*^′^) of the transitions with their decimal indices. In the case of no transition the target state is the same as the source and the decimal index not shown. The updated node of the target state is again highlighted in color. To the right of the table there is the corresponding element of the kinetic matrix *K*. For the transition *S*_*i*_→*S*_*j*_ the corresponding element is *K*_*j*,*i*_. **c** State transition graph (STG) and kinetic matrix *K* of the BN’s master equation. STG: numbers in the vertices refer to the decimal indices of the binary states of the BN’s state space *Σ*, as shown by the red-white table in panel A. Transition rates on arcs (arrows) are explained in Eq. 2. *K* is inserted into the master equation of the dynamics of probabilities, *x*(*t*), of the BN’s states as described in Eq. 3: *dx*(*t*)/*dt*=*Kx*(*t*). The colors of nonzero entries of *K* correspond to the transition rates on the STG’s arcs. Each transition rate has a separate color used both for the corresponding arc(s) of the STG and entries of the kinetic matrix *K*. As an example, the transitions from state 1 to 5 and 2 to 6 both have the transition rate *u*_*A*_ (as it is node *A* updated from 0 to 1) and these two arcs have a dark green color, same as the corresponding entries *K*_1,5_ and *K*_2,6_. The diagonal elements of *K* are equal to the sum of the off-diagonals in the given column, taken with negative sign (see Eq. 5), e.g. the entry *K*_2,2_ contains −(*u*_*A*_+*u*_*B*_). Terminal vertices that are attractor states and their corresponding columns of *K* are in gray. **d** Topological sorting of the STG in **c**. The vertices of the STG are re-indexed by topological sorting with indices ascending from upstream to downstream vertices (color coding of arcs by the transition rates does not change from panel C). This entails reordering of the kinetic matrix *K*→*K*^′^ and the columns of terminal vertices (attractor states of the BN, gray columns) being moved to the right of *K*^′^, creating a block structure (Eq. 15) used for obtaining the stationary solution (Eq. 7). **e** Construction of the right (*R*) and left (*L*) kernels from *K*^′^. *R* is a basis for the column null space of *K*^′^ and has 3 columns as there are 3 terminal vertices (6,7,8 of the topologically sorted STG). Block *Y* of *R* corresponds to non-terminal vertices, therefore it has 5 rows and contains only 0s. Block *V* corresponds to the terminal vertices, therefore it has 3 rows with 1 in the rows of terminal vertices 6,7,8. The block *U* of *L* is constructed by transposing *V* of *R* and replacing nonzero elements by 1, so that *U*·*V*=*I*. Block *X* of *L* is calculated from the blocks *B* and *N* of *K*^′^ as *X*=−*U*·*B*·*N*^−1^. The nonzero terms of *X* of *L*, *L*_*ij*_, are rational functions in the transition rates, encoding the conservations between non-terminal and terminal (attractor) states. The terms *κ*_1_,*κ*_2_ are *κ*_1_=*d*_*A*_+*d*_*B*_+*u*_*C*_, *κ*_2_=(*d*_*A*_+2*d*_*B*_+*u*_*C*_)

*V* will refer to the nodes themselves, whereas the state of the system (the values of the nodes) will be referred to as *S*. *S* is a binary number of length *n*, with each node either 0 or 1. The possible values of *S* range from [00...0]^*T*^ to [11...1]^*T*^, comprising the BN’s state space *Σ*. These binary states are ordered in the standard way and a particular state will be referred to by its decimal index *i* as *S*_*i*_. Throughout the “Methods” section we will use the 3-node BN in Fig. 1 to illustrate the concepts of the formalism. For this BN the state space *Σ* contains 2^3^=8 states ranging from *S*_1_=[000] to *S*_8_=[111], shown on Fig. 1a, where we also show the signed and directed *influence graph* of the interactions between the nodes as customary for network models [26].

When referring to the value of node *v*_*j*_ of a particular state *S*_*i*_ this will be referred to by $S_{i}^{j}$. For instance the values of nodes within state *S*_7_=110 are $S_{7}^{1}{=}1, S_{7}^{2}{=}1, S_{7}^{3}{=}0$.It is often more convenient to refer to nodes by names instead of numerical indices. The nodes of the BN in Fig. 1 are named alphabetically as *V*={*A*,*B*,*C*}, and its Boolean functions are *F*={∼*B*,(∼*A*)&*C*,*B*|*C*}, where ^′^∼^′^,^′^&^′^,^′^|^′^ are the NOT, AND, OR Boolean operators, respectively.

### Asynchronous updating

To generate a dynamical system, the Boolean functions *F* are applied to the states *S* of the system in updating steps. There are two basic types of *updating policies*, synchronous and asynchronous. In the synchronous updating formalism, all elements of *S*(*t*) are synchronously (simultaneously) updated by the Boolean functions *F* as *S*(*t*+1)=*F*(*S*(*t*)). Synchronous updating is deterministic and creates a directed graph of transitions between the states of the state space *Σ* where all states have either zero or one outgoing transition. It is also less biologically realistic as it assumes all nodes of a biological network change their state in unison, ie. they have identical timescales.

In this article we use instead the more biologically realistic *asynchronous* updating policy [27, 28], complemented by transition rates for the BN’s nodes [13], to account for differences in the timescale of activation and deactivation of the nodes. We will refer to the class of Boolean models we use as *asynchronous stochastic continuous-time logical models* (ASCTLM).

Asynchronous updating means that only one node of the BN is updated at a given time *t*, which entails that from a particular state *S* the BN can transition into multiple other states *S*^′^, depending on which node is updated. The directed graph of these transitions between all possible states *Σ* is the *state transition graph* (STG) of the BN, shown for our illustrative model in Fig. 1c. Which node *v*_*i*_ is updated at a given updating step can either be defined by transition probabilities in a discrete time framework, or by *transition rates* in a continuous time framework [13]. We will use the continuous-time framework here, as in this hybrid approach differences in the timescales of activation and deactivation of a network’s nodes can be incorporated. This provides an approximation of real physical time and the possibility to explore the dependence of attractor probabilities on these timescales.

Another stochastic Boolean framework is the probabilistic Boolean network (PBN) [25, 29] approach. The PBN approach is an ensemble modeling framework that is probabilistic due to stochastic switching between multiple possible Boolean functions per node, whereas in our ASCTLM framework stochasticity comes from stochastic asynchronous updating of the BN’s nodes (but with only one Boolean function per node). We discuss the differences of PBN in scope and formalism with our approach in SI Section 5.

### Continuous-time treatment of Boolean dynamics

Within the asynchronous updating policy time can be treated as discrete or continuous. If time is discrete then one node of *S* is updated at uniformly placed time steps according to update probabilities assigned to the nodes. Here we use the alternative *continuous time* generalization of the asynchronous updating policy. A formal derivation of the asynchronous updating policy with continuous time is provided in [13]. The main concepts are briefly outlined below.

In asynchronous updating there can be a transition between two states *S* and *S*^′^ if they differ by the value of only one node. In only this case can the *transition rate*$\phantom {\dot {i}\!}\rho _{S\rightarrow S'}$ between these two states be non-zero. If it is node *j* that differs from *S* to *S*^′^ then the transition rate is

2 $$ \begin{aligned} \rho_{S\rightarrow S'} = u_{j} \qquad if \ S^{j}=0 \\ \rho_{S\rightarrow S'} = d_{j} \qquad if \ S^{j}=1  \end{aligned}  $$

The transition rate *u*_*j*_ defines the timescale of activation and *d*_*j*_ the deactivation for node *v*_*j*_. In Fig. 1c these transition rates are the labels of the STG’s arcs. As an example, following Eq. 2, the transition [110]→[100](by decimal indices *S*_7_→*S*_5_) has the transition rate *d*_*B*_, as it is the 1→0 transition of the second node *B*. The transition [110]→[010](*S*_7_→*S*_3_) has the rate *d*_*A*_ and the transition [110]→[111](*S*_7_→*S*_8_) the rate *u*_*C*_.

Finally, we insert these transition rates into a master equation [17, 30] of the instantaneous probabilities of the BN’s states, treating their time evolution as a continuous-time Markov process. The master equation of this continuous-time Markov chain is the system of linear ordinary differential equations:

3 $$ \frac{d x(t)}{dt} = K x(t)  $$

where *x*(*t*) is a column vector of 2^*n*^ elements, with the *i*th element *x*_*i*_(*t*) being the probability that the BN occupies the *i*th binary state *S*_*i*_ of its state space *Σ*, at time *t*. Since these are probabilities, the following constraints apply for *x*(*t*):

4 $$ x_{i}(t) \in [0,1], \qquad \qquad \sum\limits_{i}^{2^{n}} x_{i}(t)=1  $$

*K* is the Laplacian matrix [22] of the directed graph of the BN’s STG, and has a dimension of 2^*n*^×2^*n*^. From here *K* will be referred to as the *kinetic matrix*, as we will use tools from chemical kinetics to obtain the stationary solution of Eq. 3 and it is mathematically equivalent to the kinetic matrix of first-order chemical reactions. The dimension of *K* will be referred to as *ν*(=2^*n*^) to avoid confusion with the number of nodes *n*. *K* and its relationship with the STG is shown for our 3-node example model in Fig. 1b.

*K* is constructed from the Boolean functions *F* and the set of transition rates {*u*_*k*_,*d*_*k*_}, *k*=1...*n* in the following steps, illustrated in Fig. 1b. The list of all transitions is generated by applying the *n* Boolean functions in *F* node-wise to the 2^*n*^ binary states of *Σ* of the model. This defines *n*×2^*n*^ possible transitions, as from any state *S*_*i*_ one out of *n* nodes can be updated. The number of actual transitions depends on the logical rules and will be smaller than (at most equal to) *n*×2^*n*^. These are shown in Fig. 1b by the color-coded arrows labeled by the transition rates between the list of source states *S* (table on the left) and target states *S*^′^ (table on the right).

When applying the Boolean function *f*_*i*_ to $S_{j}^{i}$ leads to a change in the node’s value this results in the state transition *S*_*j*_→*S*_*k*_, inserted into the entry *K*_*k*,*j*_ of the kinetic matrix. The transition will have the rate *u*_*i*_ or *d*_*i*_ as defined by Eq. 2. Identically to chemical kinetics [17, 31], the off-diagonal elements of row *i* of *K* correspond to the rates of incoming transitions to state *i*, whereas the off-diagonal elements of column *j* contain the rates of outgoing transitions from state *j*. The diagonal element *K*_*j*,*j*_ of column *j* is then equal to

5 $$ K_{j,j} = - \sum\limits_{i=1,i{\neq}j}^{2^{n}} K_{i,j}  $$

as illustrated by the color coding of the edges of the STG and the kinetic matrix in Fig. 1c.

These steps are implemented in our ExaStoLog toolbox. The user inputs a biochemical model in Boolean format (list of nodes *V* and Boolean functions *F*) and the STG and its kinetic matrix *K* are automatically generated. The default option is to assign either identical or random values to the transition rates *u*_*i*_, *d*_*i*_, *i*=1...*n*, but specific values can also be assigned when information about differing timescales is available.

With these concepts, a BN of *n* nodes is described as a continuous time ODE-system of the probabilities of its 2^*n*^ binary states. On SI Fig. 1 we show the continuous dynamics of the probabilities of the 8 states of the BN of Fig. 1a. In practice we rarely want to either analytically solve or numerically simulate the master equation because of its dimension. Instead we are interested in the *asymptotic behavior* of BNs, in other words we want to obtain the stationary solution of Eq. 3. We turn to deriving the stationary solution in the next section.

### Derivation of the stationary solution

In the discrete-time framework the stationary solution of such a system can be calculated by taking the initial values and exponentiating the system’s transition matrix, but since the dimension of the matrix grows exponentially, this is possible only for small systems [32–34], typically BNs with *n*≤10. In the context of PBNs LU decomposition was used [24] on the perturbed transition matrix to push an exact method above 20 nodes. However this was under the assumption of a reversible (ergodic) Markov chain and therefore a history-independent stationary solution. Since we want to model irreversible cell fate transitions such as cell division or death this assumption is not made here.

There is another path to an exact solution in the continuous-time framework. The master equation of an ASCTLM in Eq. 3 is a first-order, homogeneous system of linear ODEs. The sparsity of its kinetic matrix can be exploited to push the limits of an exact calculation in terms of model size, without making the model artificially reversible and consequently history-independent. In deterministic chemical kinetics a mass-action system of first order chemical reactions has identical mathematical form to the master equation of a BN shown in Eq. 3 [21, 22].

**Fig. 2:**
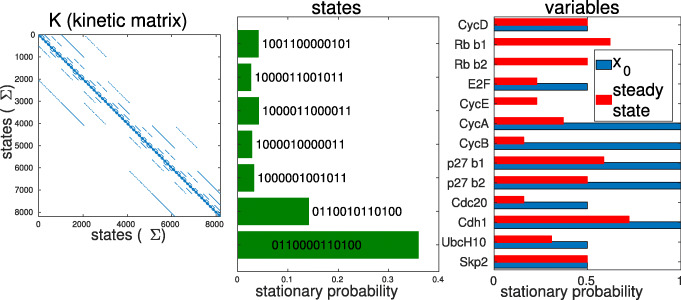
Kinetic matrix *K* and stationary solution of the mammalian cell cycle model [38]. **a** Kinetic matrix *K* from Eq. 3. The axes correspond to the 2^13^ binary states of the model. Nonzero elements are in blue. *K* has 2^26^≈7·10^7^ entries with only ≈5·10^4^ of them nonzero. **b** Stationary probability values of the model’s attractor states with >3*%* probability. The two states at the bottom are fixed points, the other states are part of the cyclic attractor. **c** Initial and stationary probabilities per model nodes

For such a linear system, as proved in [22], for any directed graph of the variables and any initial condition *x*(0), the system converges to a stable steady state (stationary solution) and this solution can be exactly calculated from the kinetic matrix *K* and the initial condition *x*(0). The master equation of a continuous-time Markov process, of which stochastic Boolean models are special examples, is mathematically identical to the rate equations of first-order chemical kinetics, having no non-linearities and hence also guaranteed to have a stable solution. While the dimension of the kinetic matrix *K* grows exponentially with the number of the model’s variables *n* as *ν*=2^*n*^, *K* is very sparse, as shown in Fig. 2a, therefore it does not need to be stored in full matrix form.

Below we reproduce the derivation of the stationary solution from [22], specifically applied to Boolean networks, graphically illustrated for the 3-node example in Fig. 1c-e.

From standard theory of linear dynamical systems [35] the solution to Eq. 3 is given by

6 $$ x(t) = exp(Kt) x(0) = (I + K{\cdot}B(t))\ x(0)  $$

where *B*(*t*) is a matrix function of time *t*.

The central finding from [22] that we use is that the stationary solution *x*^∗^=*x*(*t*) for *t*→*∞* is given from the left and right nullspaces (alternatively called kernels), *R* and *L*, of the kinetic matrix *K* as

7 $$ x^{*} = R\cdot L\cdot x(0)  $$

with the following definitions of *R* and *L*, and a normalization constraint between them:

8 $$ K\cdot R=0, \ \ L\cdot K = 0, \ \ L \cdot R = I_{q}  $$

As stated in the previous section the dimension of *K* is *ν*=2^*n*^, so if the dimension of the null space of *K* is *q*, then the right kernel matrix *R* has a dimension of *ν*×*q* and its columns are the basis of the column null space of *K*. Correspondingly, *L* is the left kernel that has a dimension of *q*×*ν*, and its rows are the basis of the row null space of *K*. *I*_*q*_ is an identity matrix of dimension *q*×*q*.

It needs to be shown that if *L* and *R* are chosen so that Eq. 8 holds, then the stationary solution is indeed given by Eq. 7.

If the constraints of Eq. 8 hold then using *L*·*K*=0 and multiplying both sides of Eq. 6 by *L* yields:

9 $$ L\cdot x(t) = \left(L\cdot I + L\cdot K\cdot B(t) \right)\cdot x(0) = (L + 0\cdot B(t))\cdot x(0) = L\cdot x(0)  $$

establishing the conservation of variables by the rows of the left kernel:

10 $$ L\cdot x(t) = L\cdot x(0)  $$

that also holds for the stationary solution *x*^∗^, so that

11 $$ L\cdot x^{*} = L\cdot x(0)  $$

When the system reaches its stationary solution *x*^∗^, this means by definition that there is no further transient dynamics so that the time derivatives of all variables are zero:

12 $$ \frac{d x^{*}}{dt} = K x^{*} = 0  $$

and *x*^∗^ is in the column null space.

Therefore *x*^∗^ is a linear combination of the columns of *R* and for some vector *d* it will hold that

13 $$ x^{*}=R\cdot d  $$

Combining Eq. 13 with the third constraint *L*·*R*=*I* from Eq. 8 and the conservation from Eq. 11 we get:

14 $$ L\cdot x(0) = L\cdot x^{*} = L\cdot (R\cdot d) = I\cdot d = d  $$

proving *L*·*x*(0)=*d*.

Then we substitute *L*·*x*(0) into *d* in *x*^∗^=*R*·*d* proving the starting assertion of Eq. 7 that *x*^∗^=*R*·*L*·*x*(0).

This proves that the stationary solution can be calculated from the left and right nullspaces of *K*, if they are chosen so that the three conditions of Eq. 8 hold. The task is therefore to build *L* and *R* accordingly, which is done by using the BN’s STG.

### Construction of the nullspaces from the state transition graph

To obtain the stationary solution we do not calculate the kernels by standard methods for two reasons. One is the dimension of the kinetic matrix, making these calculations too time consuming. Second, the zero-eigenvalue eigenvectors calculated by standard methods from *K* would satisfy the criteria *Kr*^*k*^=0 and *l*^*k*^*K*=0 (*r*^*k*^ is the *k*th column of *R*, same for *l*^*k*^ and *L*), but not the normalization condition of *L*·*R*=*I*_*q*_ and therefore yield numerically incorrect values for *x*^∗^.

*L* and *R* can be instead built from the directed graph of the STG by decomposing its kinetic matrix so that Eq. 8 holds and the nullspaces give us the correct stationary probabilities of the attractors. This procedure is described in [22] for chemical kinetics, we adopt it here to the STG of a BN. All following steps are implemented as built-in functions in our *ExaStoLog* toolbox, so that the user inputs a Boolean model and an initial condition (specifying which nodes are 0 or 1 at *t*=0) and ExaStoLog outputs the stationary probability values of the attractors. All subsequent figures are generated as results of the calculations by the toolbox.

First the vertices of the STG need to be topologically sorted [36] and the kinetic matrix *K* reordered accordingly. Topological sorting entails re-indexing the vertices of the STG (Fig. 1d) such that the index of a vertex *i* is always smaller than that of *j* if there is a directed path from *i* to *j* and no path from *j* to *i*. Multiple vertices can form strongly connected components (SCC). Within a SCC there is a directed path between any two vertices, in other words the vertices form a cycle. In terms of the BN this means a set of states that the model cycles through. Since topological sorting is only possible on an acyclic graph, it is carried out on the metagraph of the STG [37]. Therefore, the multi-vertex SCCs are treated as single vertices for this step while retaining the index of their constituent vertices. After topological sorting of the metagraph, the constituent vertices of the multi-vertex SCCs are again unmerged, with their indices having values between those of the directly upstream and downstream vertices. The indices of states internal to SCCs can retain their original binary ordering or alternatively we can apply topological sorting within multi-vertex SCCs, by breaking them into a linear chain and eventually reconnecting them, to increase the lower triangularity of the reordered kinetic matrix *K*^′^ and the matrix inversion of Eq. 18 faster. Topological sorting is shown in Fig. 1c-d, with the original STG and its kinetic matrix *K* in (c) and the relabeled STG and reordered *K*^′^ in (d). All subsequent calculations are done on the reordered kinetic matrix *K*^′^. Evidently, the original mapping between indices and the corresponding logical states needs to be retained to eventually have the correct assignment of the probability values to the logical states form the attractors. In the example in Fig. 1c-d, the attractor states with index 4,5,6 are relabeled by topological sorting as states 6,7,8 for the matrix calculations, but the stationary probabilities are eventually assigned to the original logical states *S*_4_=[011],*S*_5_=[100],*S*_6_=[101].

The STG of logical models typically have many irreversible transitions, therefore most SCCs are single vertices. In the case of our 3-node BN in Fig. 1c-d all SCCs are single vertices, the STG has no (either non-terminal or terminal) cycles.

Once this reordering is done (performed in ExaStoLog by the built-in MATLAB graph algorithm *toposort*), the reordered kinetic matrix *K*^′^ will have a block structure that is used to build the nullspaces. The terminal vertices that correspond to the attractor states of the BN are on the right of *K*^′^. It is useful here to recall the correspondence between the vertices/SCCs of the STG and the attractors of the BN. A terminal SCC of the STG corresponds to an attractor of the BN that can be either a *stable state* (also called fixed point) or a *cyclic attractor*. A stable state is made up a single state *S*, corresponding to a single terminal vertex (or sink vertex) of the STG. Cyclic attractors are made up of multiple states and correspond to terminal SCCs of multiple vertices of the STG. If the total number of non-terminal vertices is *u*, then we have *ν*−*u* vertices in the *q* terminal SCCs, corresponding to the last *ν*−*u* columns of *K*^′^. In the case of stable state attractors only, the number of terminal SCCs (number of attractors) and the number of terminal vertices (number of states in the attractors) are equal, so that *q*=*ν*−*u*. If there are cyclic attractors, there are more terminal vertices than terminal SCCs and *q*<*ν*−*u*. In either case the dimension (number of rows of row nullspace *L*, and number of columns of column nullspace *R*) of the nullspaces is *q*, equal to the number of terminal SCCs (number of attractors).

The block structure of *K*^′^ is the following, shown in Fig. 1d:


15


Here and in Eq. 16 horizontal and vertical lines show the borders between blocks and the parentheses the limits of the matrix.

*N* corresponds to the transitions within the *u* non-terminal vertices of the topologically sorted STG.

*B* corresponds to the incoming transitions of the terminal vertices.

The 0 section corresponds to outgoing transitions from terminal to non-terminal states, but since these are nonexistent it contains only zeros.

Finally, *T* corresponds to transitions between the terminal vertices. In the case of fixed point attractors there are no connections between the terminal states so this section contains zeros only, as in Fig. 1d. In the case of a cyclic attractor of multiple vertices, *T* contains nonzero elements that comprise the kinetic matrix of the transitions within the terminal cycle.

From these blocks of *K*^′^, the right (*R*) and left (*L*) kernels can be constructed step-by-step by using the structure of the STG and applying the constraints of Eq. 12. When completed, *R* and *L* will have the following block structure:

16 $$ R = \begin{aligned}\left(\frac{Y}{V} \right), \qquad L = \bigg(X\bigg|U \bigg) \end{aligned}  $$

We review how the kernels are built by defining these blocks. What is required is that finally the conditions of Eq. 8 hold. The steps are illustrated in Fig. 1e. First, the right kernel is constructed from the STG so that each column corresponds to an attractor of the BN. This entails that each column of *R* corresponds to that stationary solution where $\frac {d x^{*}}{dt}{=}K x^{*}{=}0$ and the total probability of 1 (see Eq. 4) is placed onto the vertex (vertices) of that particular attractor. Due to topological sorting the attractors have the highest index and multi-vertex terminal SCCs are identified by fast graph algorithms. This enforces the constraint *K*^′^·*R*=0 and it will also be easy to impose *L*·*R*=*I*. We now explicitly define the sections *Y* and *V* of *R* accordingly.

The section *Y* of the right kernel will correspond to non-terminal vertices and therefore has *u* rows and contains only zeros since non-terminal vertices (transient states that are not in attractors) carry 0 probability in steady state.

The section *V* will contain the states of the attractors and comprise the last *ν*−*u* rows of *R*. If vertex *i* is a stable state attractor the corresponding column of *R* contains a single nonzero element in its *i*th row. This is shown for *K*^′^ and its right kernel in Figs. 1d and e for the stable states (terminal vertices) 6,7,8: these correspond to the three columns of *R* (panel E), which have a 1 in their 6th, 7th and 8th row, respectively.

**Fig. 3:**
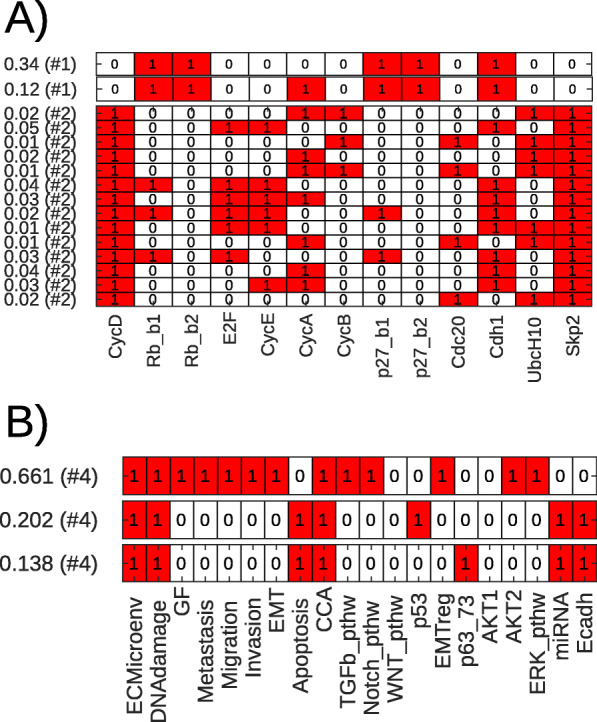
Attractors and their stationary probabilities for two models, calculation and visualization by ExaStoLog functions. Each row is an attractor state of the BN, with the columns corresponding to the BN’s nodes, listed at the bottom of the panels. The value to the left of each row is the stationary probability of the attractor state. In parenthesis is the index of the STG’s subgraph the state is located in (if there are disconnected subgraphs because of non-dynamic nodes). **a** Attractors of the mammalian cell cycle model from [38]. The two states at the top are separate stable states (fixed points) in subgraph 1. The lower 13 states are the >1*%* probability states of the cyclic attractor of 270 states. **b** Attractors of the EMT (epithelial-mesenchymal transition) model from [40]. All attractors are separate stable states

In case of a cyclic attractor the total probability of 1 is distributed among the constituent vertices according to the transition rates, so the *sum* of nonzero elements in the corresponding column of *R* is 1. These nonzero elements are calculated by taking the minors of the kinetic matrix of the cycle’s vertices, as described in [22]. A model with a large (270 constituent states) cyclic attractor is solved in Figs. 2 and 3. Like topological sorting, finding the terminal SCCs to construct the right kernel is implemented in ExaStoLog with built-in MATLAB graph theory algorithms.

To build the left kernel *L*, the constraint *L*·*R*=*I*_*q*_ is first used, which by block multiplication requires that

17 $$ U\cdot V = I_{q}  $$

To build the block *U*, *V* is transposed and its nonzero elements replaced by 1 (for fixed point attractors these are already 1). Because each column of *V* sums to 1 this satisfies *U*·*V*=*I*_*q*_ and also *L*·*R*=*I*, because the section *Y* of *R* contains only zeros.

The other constraint *L*·*K*^′^=0 has to hold too. Doing the matrix multiplication blockwise first for the section of *K*^′^ (see Eq. 15) corresponding to terminal vertices this means that *X*·0=0 and *U*·*T*=0. *X*·0=0 has to be true since the term on the right is a matrix of zeros. For stable state attractors *T* is also only zeros, so *U*·*T*=0 is again true.

For cyclic attractors, recall that the columns of the kinetic matrix sum to 0 (Eq. 5) and this is true for the columns of the section $\left (\frac {0}{T}\right)$ of *K*^′^ too. A column of *U* contains 1s for the rows of all the vertices of a terminal cycle and a column of *T* contains the outgoing transitions of one of the cycle’s vertices and a diagonal element (Eq. 5) that sum to 0. *U* sums these entries of *T* therefore *U*·*T*=0 is true for the case of a cyclic attractor too, so *U*·*T*=0 holds for any attractor type.

The only constraint that still needs to be respected is to have the remaining block of the left kernel *X* so that *L*·*K*^′^=0 is satisfied. This requires by block multiplication that *X*·*N*+*U*·*B*=0 and since section *N* of *K*^′^ is always invertible due to topological sorting, the missing part *X* of the left kernel *L* can be obtained as:

18 $$ X{=}-U \cdot B \cdot N^{-1}  $$

Because of topological sorting *N* is a lower triangular matrix if the STG contains no cycles, and contains few elements in its upper triangular section if there are small non-terminal cycles. Therefore, its inversion is a fast calculation. If the STG contains large (more than a thousand vertices) non-terminal cycles, the inversion is more time-consuming. For the models in Table 1 we have not confronted this problem. The calculation of *X* is shown graphically in Fig. 1e, with the nonzero terms of *X* in symbolic form. *X* contains rational functions in the model’s transition rates, which encode the conservations between the model’s non-terminal states and attractors. Mathematically they are ratios of polynomials with (only) positive coefficients, originating from the forks and cycles of the STG that distribute the initial probabilities on the vertices into the attractor states. As shown by Fig. 1e even for a 3-node model the denominators contain quadratic terms and for larger models contain polynomials of high order, showing that the dependence of stationary solutions on transition rates is a complex mathematical expression. In summary, up to the limit that we can store the transitions of a logical model as a sparse matrix its stationary solution can be obtained by topological sorting of its state transition graph and matrix calculations on its reordered kinetic matrix. Using our ExaStoLog toolbox, for biological models of 13-23 variables the calculation of the stationary solution is of the order of seconds, and the memory requirement for storing the kinetic matrix exceeds 1GB at 23 nodes.

**Table 1:** Properties of Boolean models analyzed in manuscript

**# Model [ref]**	**nodes (dynamic)**	**Matrix (Mbytes, density)**	**calculation time**
#1 [38]	13 (12)	0.9Mb, 7e-4	0.35-0.6 sec
#2 [39]	20 (16)	143Mb, 7e-6	0.4-3 sec
#3 [40]	20 (18)	173Mb, 8e-6	2.5-25sec
#4 [41]	20 (19)	173Mb, 9e-6	2-19 sec
#4.1 [39]	23 (19)	1.38Gb, 1e-6	2-30 sec
#4.2 [39]	23 (21)	1.44Gb, 1e-6	13-43 sec

Calculation time is for solving a model with one set of initial conditions and transition rates on a computer with 8 cores (Intel(R) Xeon(R) CPU X5472 3.00GHz), without parallelization. In parenthesis in the column ‘nodes’ are the numbers of dynamic nodes, excluding input nodes.

## Results

### ExaStoLog toolbox: calculation of solutions, visualization and parameter sensitivity analysis

The above steps of calculating the stationary solution of a logical model are implemented in the MATLAB toolbox *ExaStoLog*, available on GitHub with a detailed tutorial [23]. The user can input a logical model in *BoolNet* [12] format using standard logical notation. The generation and topological sorting of the STG and the identification of its cycles are steps independent of the values of the transition rates, therefore these are performed only once for a given model. The STG is stored as a ‘cell’ with the indices, but not the numerical values of the transition rates. The kinetic matrix containing the numerical values of the transition rates is stored in the sparse matrix format of MATLAB. The time of calculation and the memory requirement for storing *K* are shown in Table 1 for four different models that we analyzed.

The STG of the [39] model (both in its 20 and 23 node version) contains no cycles, therefore its solution is fast to calculate as its *K*^′^ is completely lower triangular. The other models contain cycles of up to a few hundred (in one case more than a thousand) vertices, reflected in the calculation time. To test the upper bound of the current implementation of the toolbox, we have also run ExaStoLog on two 23-node versions of this same model, shown in the last two rows of Table 1. All references to the model in [39] are to the 20-node version.

Biological models often have input nodes that are not dynamic, representing environmental conditions such as the presence of a drug or extracellular ligand. Such models have STGs made up of disconnected subgraphs. In this case, the time of calculation also depends on how many subgraphs contain states with nonzero initial probability.

Besides the calculation of the stationary solution, ExaStoLog contains 16 functions to visualize the results and to perform parameter sensitivity analysis and parameter fitting.

All figures in the main text (except Fig. 1) and all SI Figs. (except 1, 3-4, 6-7) were generated by the functions of ExaStoLog.

To ensure reproducibility of the results, we compared them for multiple models, both with separate stable states and cyclic attractors, to Monte Carlo simulations and verified that the results are identical, as shown on SI Figs. 1 and 2.

We also compared runtime requirements for stochastic simulations (performed by MaBoSS [13]) and exact calculations, shown in Table 2.

**Table 2:** Runtime comparisons of stochastic simulations (MaBoSS) and ExaStoLog

**Model**	**nodes**	**# param. sets (*n***^***p***^**)**	**MaBoSS runtime**	**ExaStoLog**
*kras15* [23]	15	729 (3^6^)	4mins20s	19s (2s)
[39]	20	729 (3^6^)	33mins	8mins (1min)

Calculations on the same computer as Table 1 (single core). The *kras15* model is by the authors, available at [23] in the *model_files* folder, together with input files for the parameter scans for both models. The *kras15* model was run with 10.000 trajectories for a duration of 3 time units, time steps of 0.1. The [39] model was run with 20.000 trajectories, for 40 time units, time steps of 0.1. In both cases an accuracy of 1% was imposed as a requirement. In parenthesis in the column ExaStoLog is the amount of time spent on the stationary solution calculations themselves.

The results of these comparisons will vary with the structure of the Boolean model. The two models ([39] and a 15-node model developed by us, available at [23]) selected for the comparison have STGs without large non-terminal cycles. This choice was made as large non-terminal cycles in the STG are difficult to interpret biologically, and they can also distort the results of stochastic simulations as well as slow down the matrix inversion for the exact solution. To make the results comparable, we have set the number of trajectories and duration of stochastic simulations so that the deviation of the stationary probabilities from the exact result does not exceed 1%. The parameter sampling with MaBoSS is by an efficient C++ code available at [42]. With these settings we performed multidimensional parameter scans with *p* transition rates and *n* sampling values, i.e. for *n*^*p*^ parameter sets. For the [39] model the six transition rates with the highest Sobol sensitivity index were selected for the parameter scan. The values were logarithmically distributed (0.1, 1, 10). The results are shown in Table 2. For the model with 15 nodes the exact method is approximately an order of magnitude faster than stochastic simulations. For the 20-node [39] model for 1% accuracy the duration of the simulation and the number of samples had to be significantly increased (see Table 2). This is because for more than half of parameter sets non-terminal states linger on and have nonzero probabilities at the end of the simulations. Note that when relying on Monte Carlo simulations it is not known in advance what trajectory number and simulation time is required for convergence. Adaptive methods for convergence can address this problem, but the parameter scan for the [39] model shows that at certain transition rate values non-terminal states can survive for a long time and distort estimates. The exact method does not require experimentation with the number of sample trajectories and the duration of simulations to ensure all attractors are found and their probability well-estimated. Approximately 90% of total calculation time for the exact method is spent in regenerating the kinetic matrix with the changed parameter values and only around 10% on calculating the stationary solutions (see Table 2). In summary, when the aim is to mechanistically understand relatively small models by parameter sensitivity analysis, the exact calculation is an efficient way to do it, more so than stochastic simulations.

Besides the advantage in speed and precision, in *ExaStoLog* there is an environment of a dozen functions for visualization and analysis of the results of sensitivity analysis. The parametric analysis of the probability of attractor states with regard to transition rates and the visualization of such analysis is currently not available in other Boolean modeling platforms, such as GinSim [11], BoolNet [12] or MaBoSS [13].

Below we discuss the results obtained by ExaStoLog’s functions for parameter sensitivity analysis for the four published Boolean models listed in Table 1.

### Application to published biological models

The exact method in its current implementation is best-suited to study the stationary solutions and identify the key parameters (transition rates) of logical models of intermediate size, the largest model analyzed in this article is 23 nodes (see Table 1). We selected four models, listed in Table 1, from the literature to illustrate the exact method and the sensitivity analysis and parameter fitting features of ExaStoLog. All plots are with models 1-4 of Table 1. The last two rows of Table 1 refer to expanded versions of the [39] model (4.1, 4.2) to test the limit for model size, but they are not analyzed further.

The models are described in more biological detail in SI Section 2, here we give only a very brief summary: [38] presents a discrete model of the mammalian cell cycle. The model in [39] describes signaling pathways involved in breast cancer and focuses on resistance mechanisms. The model in [40] explores the dynamics of the early steps of the metastatic process. Finally, [41] is a Boolean model of breast cancer with an emphasis on ERBB2 overexpression. The model of mammalian cell cycle is known to have a cyclic attractor, the others are expected to have fixed point attractors.

We pose the question to what extent the behavior of these models are parameter-dependent and if parameter sensitivity analysis can aid model reduction, similarly to chemical kinetics [31].

All the visualizations below were generated by first calling the functions of ExaStoLog that implement the calculations described in *Methods* and subsequently the functions designated for the visualization of results. The examples shown can be reproduced with the tutorial and the input files at [23]. First, we visualize the model’s attractors (Fig. 2b and Fig. 3) and their corresponding probabilities.

Some models have many attractors and/or states in attractors, eg. the breast cancer model [39] has 39 fixed points, as shown on SI Fig. 5. The mammalian cell cycle model [38] has a large cyclic attractor of 270 states. For these cases, projecting the attractor probabilities onto the model nodes is more biologically informative, shown in Fig. 2c for [38] and SI Fig. 5 for [39]. This is done by multiplying the binary numbers of the attractor states with their respective stationary probabilities and taking their sum. This yields stationary probabilities by model nodes (referred to as ‘node probability’) which can be interpreted as probabilities that a particular node of the logical model is activated.

We want to identify transition rates that are more important in defining the model’s behavior, which can be for instance the stationary probability of model nodes representing biomarkers or phenotypes. We first gauge parameter sensitivity by one-dimensional parameter scans of all transition rates, from which those where model variables show significant variation can be selected.

**Fig. 4:**
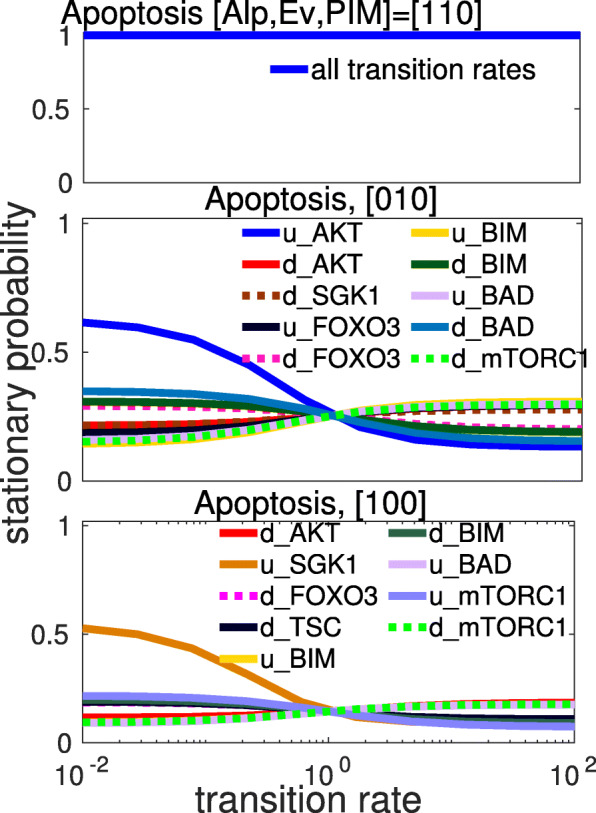
Effect of one-dimensional parameter scan on the stationary probability (y axis) of the *Apoptosis* node of the breast cancer model[39]. The stationary probability value of the *Apoptosis* node is plotted as a function of the transition rates in the legends. Only transition rates where the variation of at least one node’s stationary value is larger than 0.1 are shown. The three panels show the model’s behavior under 3 different settings of the input nodes: the drugs Alpelisib and Everolimus and the node *PIM* (PIM protein family), abbreviated [Alp,Ev,PIM]. The values [110],[010],[100] above the panels show the values of these three nodes. Under double inhibition, shown on the top panel, the model has a single attractor state with *Apoptosis=1*, irrespective of the value of transition rates

For the [39] model of drug effects in breast cancer, the sensitivity analysis showed that transition rates have differential effects as a function of the value of input nodes. Specifically, as shown in Fig. 4, in the presence of one of the two drugs (and the node *PIM*=0, standing for PIM kinases [43]), it is the transition rates *u*_*AKT*_ of the *AKT* node (middle panel) and *u*_*SGK*1_ of *SGK1* (bottom panel) that has, respectively, the most potent effect on the *Apoptosis* node. The presence of both drugs (top panel), in contrast, invariably leads to 100% apoptosis, a robust feature of this model.

**Fig. 5:**
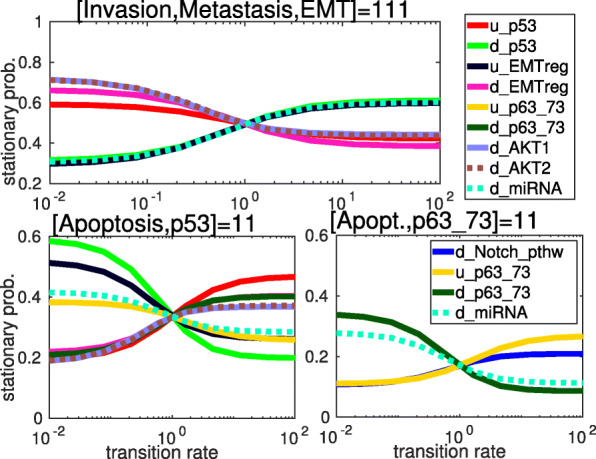
Effect of one-dimensional parameter scans on the stationary probability (y axis) of the 3 model attractors of the EMT model [40]. Transition rates included in the scan are shown in the legends. For the values of nodes within the attractor states see Fig. 3b. The curves in the top panel and the lower left panel are parameter scans in the same transition rates. The input nodes *ECMicroenv* and *DNAdamage* were set to 1 at *t*=0. The top panel is the attractor with the nodes [*Invasion, Metastasis, EMT*]=[111]. In the lower left attractor [*Apoptosis, p53, p63_73*]=[110]. In the lower right attractor [*Apoptosis, p53, p63_73*]=[101]

In Fig. 5 we show the effect of parameter scans on the stationary probabilities of the attractors (fully shown in Fig. 3b) of the [40] model on the early steps of metastasis. The top panel shows the change in the stationary probability of the attractor corresponding to the EMT-metastasis-invasion process (with the nodes *[Invasion, Metastasis, EMT]=[111]*) as a function of the value of its most sensitive transition rates. The bottom panels show in a similar way the stationary probabilities of the two attractors corresponding to the apoptotic phenotype, distinguished by the alternative activation of p53 (lower left panel) or p63/p73 (lower right). Here, the analysis shows that the decision point between the model’s proliferative-invasive and apoptotic behavior mainly depends on the transition rates of the nodes *p53, p63_73, AKT1, AKT2, Notch_pathway* and *miRNA*.

For the cell cycle model we found (SI Fig. 9) that the distribution of probabilities between the two fixed points is determined only by the transition rates ${u_{Rb\_b2}}$ (the node *Rb_b2* stands for the higher activation level of the retinoblastoma gene) and $d_{p27\_b2}$ (similarly for the p27 gene). Of the 270 states of the cyclic attractor, only 13 show a significant sensitivity to transition rates. Interestingly, each of these parameter-sensitive states has a single transition rate that can increase its probability up to 40%.

We also found an example of a model being robust to relative changes in transition rates: the breast cancer model of [41] has only one attractor state (a fixed point) if all transition rates are nonzero. It is only knockdowns of the model nodes *CDK6, CyclinD1* or *CDK4* that make it possible to access the model’s other fixed point, where *pRB*=0, which means that cell cycle progression (G1/S transition) is blocked. The effects of the different knockdowns for this model is shown in SI Fig. 8.

One-dimensional parameter scanning only covers a small subspace of a multidimensional parameter space along its axes. To extend our analysis, the transition rates that have a significant effect on stationary solutions are selected from one-dimensional parameter analysis and their multidimensional parameter space is explored using the Latin Hypercube Sampling (LHS) [19, 44] function of ExaStoLog.

The results of LHS are first visualized on scatterplots as shown on SI Fig. 12 with a trendline showing if a node’s (or state’s) stationary probability has a clear trend as a function of particular transition rates.

**Fig. 6:**
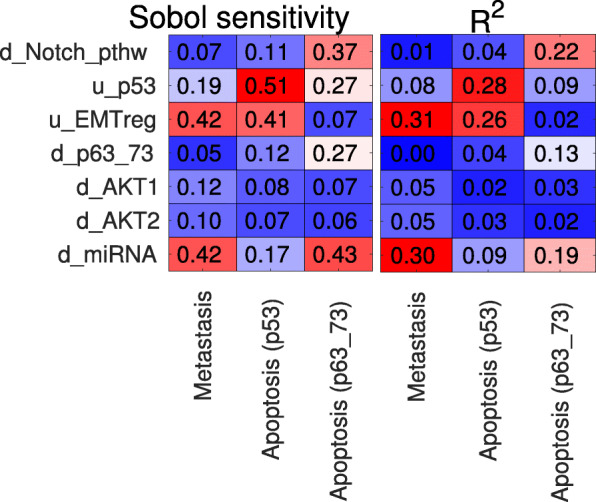
Global sensitivity of stationary attractor probabilities (x axis) with regard to transition rates (y-axis) of the EMT model [40]. The values were calculated from Latin Hypercube Sampling with 2000 parameter sets. Rows correspond to the transition rates on the y-axis; columns to the three attractor states of the EMT model (also shown on Figs. 3b and 5). The left panel shows the Sobol sensitivity indices (see SI Section 3.3) for the transition rates, the sample size was 1000 parameter sets for each resampling. The right panel shows the coefficient of determination (*R*^2^) from linear regression between transition rates and the probability values of the three attractors

Beyond this visual intuition, the effect of transition rates is statistically analyzed by performing linear regression on the stationary values by the rates, the *R*^2^ (coefficient of determination) values shown in Fig. 6 (right panel) for the EMT model’s [40] three attractor states. For this model, the transition rates of *p53*, and of the nodes representing EMT pathway regulators (*EMT_reg*) and regulatory miRNAs (*miRNA*) have the strongest explanatory value for the stationary solutions.

We can also calculate correlations between the stationary probabilities of a model’s *nodes*, to see if there are strong correlations between some. Eg. for the EMT model, the dominant upstream variables of the *Apoptosis* node are *p53, p63_73, miRNA* and *Ecadh*. Also, some nodes always have identical values which can be used for model reduction.

Performing linear regression on the stationary values by the transition rates assumes a monotonic relationship between these, which is not necessarily the case. Sobol sensitivity index [44] is a global sensitivity measure without this assumption that quantifies the relative amount of variance in a variable’s value due to variation of the individual parameters.

Calculation of the Sobol sensitivity index (described in the SI), requires recalculating the solutions by individually replacing columns of the matrix of parameters generated by LHS. In the case of the EMT model [40], shown in Fig. 6 (left panel) the Sobol sensitivity indices show a similar pattern as *R*^2^, since there are no non-monotonic effects. We have not found examples of non-monotonic effects for the models in Table 1. Finally, if quantitative data is available for a model’s variables or states it is possible to perform parameter fitting with ExaStoLog on the model’s transition rates. If a model’s nodes are proteins, quantitative phosphoprotein data is an ideal data type, often used in another semi-mechanistic modeling approach, modular response analysis [45, 46]. As described above, the stationary solutions are complex rational functions (ratios of polynomials) of transition rates (Fig. 1c), therefore it is not computationally practical to calculate gradients for parameter fitting for any model larger than a few variables. For this reason, we integrated a gradient-free simulated annealing algorithm into our toolbox (see SI Section on parameter fitting).

**Fig. 7:**
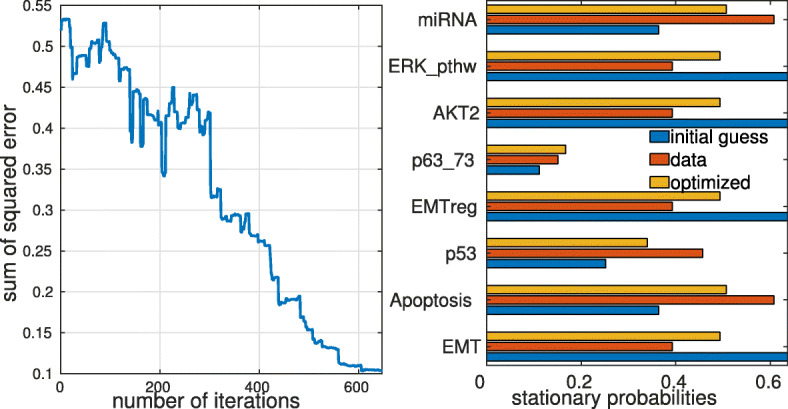
Parameter fitting of transition rates by simulated annealing for the EMT model [40]. The stationary solution of the EMT model was calculated with randomly chosen values for transition rates and simulated annealing was run on 9 transition rates of the model, started from a different (random) initial condition. The panel on the left shows the convergence process, with the y-axis showing the SSE between the stationary solution with the chosen parameters and the fitted parameters during the annealing (fitting) process. The panel on the right shows the stationary solutions with the chosen parameters (data), the initial guess and the fitted values

Fitting with simulated annealing of the 20-node EMT model is shown in Fig. 7. For some models using the initial numerical gradient of the error with respect to the fitted transition rates can be sufficient to perform parameter fitting, with less iterations and therefore lower computation time than for simulated annealing. This method can diverge as it is based on an initial estimate of the gradient. The convergence process for the two fitting methods in the case of the EMT model [40] is shown in Fig. 7 and SI Fig. 16, respectively.

With this exact method, it is therefore possible to use stochastic Boolean models for quantitative modeling and connect models to experimental data directly.

## Discussion

We have shown above that it is possible to calculate the stationary solutions of continuous time stochastic Boolean models by an exact method without Monte Carlo approximations. Additionally, this exact-continuous calculation method made it easier to explore the question of parameter dependence of this class of models. The examples of Boolean models from the literature have shown that transition rates can indeed have a significant effect on a model’s behavior and typically it is a small subset of all the rates that dominate a model’s behavior, providing a better mechanistic understanding of the model.

These findings can be used in several ways. One is the parameterization (instantiation) of models by data, as done in [18] to improve the clinically relevant predictive power of Boolean models. In this approach, transition rates can be set to 0 for knockout mutants or their values adjusted based on continuous omics data. Subsequently the phenotypic changes in model behavior can be compared to clinical data to validate the model. *Vice versa*, if data is available on the relative activation level of a model’s nodes (or relative frequency of its states corresponding to phenotypes) it can be used to fit transition rates directly.

For example, phosphoprotein measurements with perturbations [45, 46] can be used to fit transition rates, as it was done with simulated data in Fig. 7. While transition rates are not biochemical constants, they have a plausible biological interpretation as proxies for the timescale of activation-deactivation processes. Similarly, differences in their values across different cell lines or other biological samples can be interpreted as indications for corresponding differences in expression or activity levels of genes, proteins or higher level cellular processes.

## Conclusion

Stochastic logical models represent a powerful framework to study the behavior of cellular networks in terms of their steady state (attractor) behavior and their sensitivity to perturbations. An extension of asynchronously (stochastically) updated, discrete time logical models emerged in recent years [13, 14] using timescale parameters (transition rates) for the model’s nodes to generate continuous time Monte Carlo simulations. This approach provides continuous values for attractor probabilities and a model’s variables, enabling more quantitative analysis and comparison with continuous biological data.

We took this framework of analysis but implemented it with an exact method adopted from chemical kinetics to perform robustness analysis of existing models. Our analysis confirmed the possibility of efficiently applying exact methods in the context of stochastic logical models, as well as the importance of their parametric analysis.

This analysis raises several questions that call for further investigation. For the efficiency of parameter fitting and to identify a global minimum, the dependence of the stationary solution on transition rates needs to be analyzed in general form.

Currently our method has a limit in terms of model size due to the memory requirements of storing the model’s kinetic matrix (Table 1). The first way to mitigate this is to store only those states of the STG that are accessible from the initial condition. This is partially implemented in ExaStoLog inasmuch as the STG’s empty subgraphs are not used for calculations, but inaccessible individual states are not eliminated.

More fundamentally, the symmetries of the STG need to be exploited. Existing methods to simplify the STG, such as hierarchical transition graphs [27], could be integrated in our exact framework. Model simplification methods from the probabilistic model checking tool PRISM [47] for continuous-time Markov chains, such as symmetry reduction [48], can also be potentially used. These are parameter-independent steps to be performed only once for a given model, after which parameter-dependent calculations can be performed more efficiently.

Additionally, in chemical kinetics when rate constants (equivalent to our transition rates in the case of first-order chemical reactions) are well-separated this is used to approximate the eigenvectors of the kinetic matrix [49, 50]. By this method we can address dynamic behavior in a simplified form by calculating the leading eigenvalue of a model to obtain the relaxation time of the system.

By using these simplifications we intend to push the limits of our exact method to encompass larger logical models in the future.

## Supplementary information

**Additional file 1** Supplementary Information containing details of the analyzed biological models, sensitivity analysis and parameter fitting.

## Data Availability

The datasets generated and/or analysed during the current study are available in the GitHub repository, at https://github.com/sysbio-curie/exact-stoch-log-mod. No experimental data was used for this article. All calculations shown in the text can be reproduced by the toolbox at the cited repository, where a detailed tutorial is available.

## Abbreviations

BN: Boolean network
ASCTLM: Asynchronous stochastic continuous-time logical model
STG: State transition graph
SCC: Strongly connected component
EMT: Epithelial-mesenchymal transition

## References

[1] Alon U (2006). An Introduction to Systems Biology: Design Principles of Biological Circuits.

[2] Le Novere N (2015). Quantitative and logic modelling of molecular and gene networks. Nat Rev Genet.

[3] Calzone L, Barillot E, Zinovyev A (2018). Logical versus kinetic modeling of biological networks: applications in cancer research. Curr Opin Cell Eng.

[4] Aldridge BB, Saez-Rodriguez J, Muhlich JL, Sorger PK, Lauffenburger DA (2009). Fuzzy logic analysis of kinase pathway crosstalk in TNF/EGF/insulin-induced signaling. PLoS Comput Biol.

[5] Wynn ML, Consul N, Merajver SD, Schnell S (2012). Logic-based models in systems biology: a predictive and parameter-free network analysis method. Integr Biol.

[6] Morris MK, Saez-Rodriguez J, Sorger PK, Lauffenburger DA (2010). Logic-based models for the analysis of cell signaling networks. Biochemistry.

[7] Kauffman S (1974). The large scale structure and dynamics of gene control circuits: an ensemble approach. J Theor Biol.

[8] Kauffman SA (1969). Metabolic stability and epigenesis in randomly constructed genetic nets. J Theor Biol.

[9] Kauffman S (1969). Homeostasis and differentiation in random genetic control networks. Nature.

[10] Naldi A, Hernandez C, Abou-Jaoudé W, Monteiro PT, Chaouiya C, Thieffry D (2018). Logical modeling and analysis of cellular regulatory networks with ginsim 3.0. Front Physiol.

[11] Gonzalez AG, Naldi A, Sanchez L, Thieffry D, Chaouiya C (2006). GINsim: a software suite for the qualitative modelling, simulation and analysis of regulatory networks. Biosystems.

[12] Müssel C, Hopfensitz M, Kestler HA (2010). BoolNet?an R package for generation, reconstruction and analysis of Boolean networks. Bioinformatics.

[13] Stoll G, Viara E, Barillot E, Calzone L (2012). Continuous time Boolean modeling for biological signaling: application of Gillespie algorithm. BMC Syst Biol.

[14] Stoll G, Caron B, Viara E, Dugourd A, Zinovyev A, Naldi A, Kroemer G, Barillot E, Calzone L (2017). MaBoSS 2.0: an environment for stochastic Boolean modeling. Bioinformatics.

[15] Gillespie DT (1977). Exact stochastic simulation of coupled chemical reactions. J Phys Chem.

[16] Rao CV, Arkin AP (2003). Stochastic chemical kinetics and the quasi-steady-state assumption: Application to the Gillespie algorithm. J Chem Phys.

[17] Érdi P, Tóth J (1989). Mathematical Models of Chemical Reactions: Theory and Applications of Deterministic and Stochastic Models.

[18] Béal J, Montagud A, Traynard P, Barillot E, Calzone L (2019). Personalization of logical models with multi-omics data allows clinical stratification of patients. Front Physiol.

[19] Zi Z (2011). Sensitivity analysis approaches applied to systems biology models. IET Syst Biol.

[20] Fröhlich F, Kaltenbacher B, Theis FJ, Hasenauer J (2017). Scalable parameter estimation for genome-scale biochemical reaction networks. PLoS Comput Biol.

[21] Gunawardena J (2012). A linear framework for time-scale separation in nonlinear biochemical systems. PloS ONE.

[22] Mirzaev I, Gunawardena J (2013). Laplacian dynamics on general graphs. Bull Math Biol.

[23] Koltai M. ExaStoLog tutorial. https://github.com/sysbio-curie/exact-stoch-log-mod/tree/master/doc, Accessed 22 February 2020.

[24] Li W, Cui L-B, Ng MK (2012). On computation of the steady-state probability distribution of probabilistic Boolean networks with gene perturbation. J Comput Appl Math.

[25] Trairatphisan P, Mizera A, Pang J, Tantar AA, Schneider J, Sauter T (2013). Recent development and biomedical applications of probabilistic Boolean networks. Cell Commun Signal.

[26] Fages F, Soliman S. From reaction models to influence graphs and back: a theorem. In: International Workshop on Formal Methods in Systems Biology. Springer: 2008. p. 90–102.

[27] Bérenguier D, Chaouiya C, Monteiro PT, Naldi A, Remy E, Thieffry D, Tichit L (2013). Dynamical modeling and analysis of large cellular regulatory networks. Chaos: An Interdiscip J Nonlinear Sci.

[28] Stoll G, Rougemont J, Naef F (2006). Few crucial links assure checkpoint efficiency in the yeast cell-cycle network. Bioinformatics.

[29] Shmulevich I, Dougherty ER, Kim S, Zhang W (2002). Probabilistic Boolean networks: a rule-based uncertainty model for gene regulatory networks. Bioinformatics.

[30] Van Kampen NG (1992). Stochastic processes in physics and chemistry.

[31] Radulescu O, Gorban AN, Zinovyev A, Lilienbaum A (2008). Robust simplifications of multiscale biochemical networks. BMC Syst Biol.

[32] Brun M, Dougherty ER, Shmulevich I (2005). Steady-state probabilities for attractors in probabilistic Boolean networks. Signal Process.

[33] Zhang S-Q, Ching W-K, Ng MK, Akutsu T (2007). Simulation study in probabilistic Boolean network models for genetic regulatory networks. Int J Data Min Bioinforma.

[34] Ching W-K, Zhang S, Ng MK, Akutsu T (2007). An approximation method for solving the steady-state probability distribution of probabilistic Boolean networks. Bioinformatics.

[35] Hirsch MW, Devaney RL, Smale S (1974). Differential equations, dynamical systems, and linear algebra.

[36] Kahn AB (1962). Topological sorting of large networks. Commun ACM.

[37] Dasgupta S, Papadimitriou CH, Vazirani UV (2008). Algorithms.

[38] Traynard P, Fauré A, Fages F, Thieffry D (2016). Logical model specification aided by model-checking techniques: application to the mammalian cell cycle regulation. Bioinformatics.

[39] Zañudo JGT, Scaltriti M, Albert R (2017). A network modeling approach to elucidate drug resistance mechanisms and predict combinatorial drug treatments in breast cancer. Cancer Converg.

[40] Cohen DP, Martignetti L, Robine S, Barillot E, Zinovyev A, Calzone L (2015). Mathematical modelling of molecular pathways enabling tumour cell invasion and migration. PLoS Comput Biol.

[41] Sahin Ö, Fröhlich H, Löbke C, Korf U, Burmester S, Majety M, Mattern J, Schupp I, Chaouiya C, Thieffry D (2009). Modeling ERBB receptor-regulated G1/S transition to find novel targets for de novo trastuzumab resistance. BMC Syst Biol.

[42] Noel V. MaBoSS-Sampling. https://github.com/sysbio-curie/MaBoSS-Sampling/, Accessed 6 March 2020.

[43] Santio NM, Landor SK-J, Vahtera L, Ylä-Pelto J, Paloniemi E, Imanishi SY, Corthals G, Varjosalo M, Manoharan GB, Uri A (2016). Phosphorylation of Notch1 by Pim kinases promotes oncogenic signaling in breast and prostate cancer cells. Oncotarget.

[44] Constantine PG, Diaz P (2017). Global sensitivity metrics from active subspaces. Reliab Eng Syst Saf.

[45] Dorel M, Klinger B, Gross T, Sieber A, Prahallad A, Bosdriesz E, Wessels LF, Blüthgen N (2018). Modelling signalling networks from perturbation data. Bioinformatics.

[46] Klinger B, Sieber A, Fritsche-Guenther R, Witzel F, Berry L, Schumacher D, Yan Y, Durek P, Merchant M, Schäfer R, et al. Network quantification of EGFR signaling unveils potential for targeted combination therapy. Mol Syst Biol. 2013; 9(1). 10.1038/msb.2013.29 PMC3964313 23752269

[47] Kwiatkowska M, Norman G, Parker D (2009). PRISM: probabilistic model checking for performance and reliability analysis. ACM SIGMETRICS Perform Eval Rev.

[48] Kwiatkowska M, Norman G, Parker D. Symmetry reduction for probabilistic model checking. In: International Conference on Computer Aided Verification. Springer: 2006. p. 234–48.

[49] Gorban AN, Radulescu O (2008). Dynamic and static limitation in multiscale reaction networks, revisited. Adv Chem Eng.

[50] Gorban AN, Radulescu O, Zinovyev AY (2010). Asymptotology of chemical reaction networks. Chem Eng Sci.

